# Can Environmental Education Actions Change Public Attitudes? An Example Using the Pond Habitat and Associated Biodiversity

**DOI:** 10.1371/journal.pone.0154440

**Published:** 2016-05-05

**Authors:** Eunice Sousa, Victor Quintino, Jael Palhas, Ana Maria Rodrigues, José Teixeira

**Affiliations:** 1 CIIMAR—Interdisciplinary Center of Marine and Environmental Research, Porto, Portugal; 2 CIBIO/InBIO–Research Center in Biodiversity and Genetic Resources, Associated Laboratory, Porto, Portugal; 3 Department of Biology & CESAM–Research Center Environmental and Marine Research, University of Aveiro, Aveiro, Portugal; The Scripps Research Institute Scripps Florida, UNITED STATES

## Abstract

Ponds provide vital ecological services. They are biodiversity hotspots and important breading sites for rare and endangered species, including amphibians and dragonflies. Nevertheless, their number is decreasing due to habitat degradation caused by human activities. The “Ponds with Life” environmental education project was developed to raise public awareness and engagement in the study of ponds by promoting the direct contact between the public and nature, researchers and pedagogical hands-on exploration activities. A pre-post- project survey was set-up to assess the effects of the project on the environmental consciousness, knowledge and attitude changes towards ponds and the associated biodiversity of school students aged 15 to 18. The survey questions were based on Likert scales and their pre-post project comparisons used an innovative multivariate hypothesis testing approach. The results showed that the project improved the students’ knowledge and attitudes towards ponds and associated biodiversity, especially the amphibians. Ponds can be found or constructed in urban areas and despite small sized, they proved to be interesting model habitats and living laboratories to foster environmental education, by encompassing a high number of species and a fast ecological succession.

## Introduction

Ponds are small shallow water bodies, natural or artificial, permanent or temporary and characterized by an accentuated seasonal pattern of the water level or hydroperiod [1–5]. They exist in all continents and are considered biodiversity hotspots due to their importance as breading sites for amphibians, dragonflies and other invertebrates, as well as key habitats for diverse fauna and aquatic plants [1, 2, 5, 6]. They can harbour more species than lakes, rivers, streams and other freshwater ecosystems, as well as unique and rare taxa [7]. Mediterranean temporary ponds, in particular, have many endemic species and are protected by the directive 92/43 CEE [8], by European Commission Natura 2000 network (habitat 3170) and by the Ramsar Convention on Wetlands [1, 3–5, 9, 10].

Despite their biodiversity and ecological services, the number of ponds is decreasing, especially in the Mediterranean region [10]. Ponds are usually neglected by the public and are very susceptible to degradation, caused namely by intensive agriculture and urban development [2, 3, 6, 10]. Portugal is no exception and despite of natural climatic and geomorphological characteristics that favor the occurrence of natural ponds, including Mediterranean temporary ponds, local studies indicated an accentuated loss of this habitat and its associated biodiversity [10–13].

There is a high conservation concern for amphibians given that nearly one-third of the species (32.4%) are globally threatened [10, 14]. Many authors have documented the link between habitat loss, namely breeding sites, and amphibian decline and extinction [15–17]. Habitat change is globally the major contributing factor to amphibian decline, affecting around 87% of the threatened species [18]. Amphibians are also among the least appreciated vertebrates by the general public, often due to erroneous negative values and misconceptions from interpretations of folklore and ancient myths [19].

Biodiversity loss is one of the main concerns of the scientific community and constitutes an important issue of the educational curricula in many countries. Many researchers emphasized the importance of outdoor activities with a biodiversity and ecological educational strategy in order to develop concepts, construct attitudes, and the overall personality [20, 21]. Direct contact with biodiversity and a better understanding of its importance and threats are essential to raise public awareness and engagement in community-driven biodiversity conservation and monitoring programs. However, most of the population lives in urban areas and have decreasing direct contact with nature, limiting the efficacy of education towards environmental and biodiversity awareness [22]. From this point of view, hands-on activities in proximity habitats may help to overcome this gap by providing experiences to students, enhancing their literacy and their active participation in conservation.

“Ponds with Life” (“Charcos com Vida”) is an environmental education project developed in Portugal with the purpose of raising public awareness and engagement in the study and pedagogical exploitation of ponds and the conservation of associated biodiversity. The project details, general information about pond importance, construction, management and biodiversity, a set of pedagogical activities as well as the first nationwide pond survey can be obtained in the project website (www.charcoscomvida.org).

The sub-project “Choose Science–Ponds with Life” was designed for 15–18 year old high-school students. It included activities throughout a school year allowing a direct contact with ponds, their biodiversity and with researchers. The program featured at least five visits of one member of the “Ponds with Life” team during a school year (2013/2014) and the development of several activities, including pond adoption or construction in the school area or neighborhood, scientific lectures and workshops as well as hands-on experimental activities in the classroom, the laboratory and the field, associated to the biological monitoring of the adopted pond. In addition, an amphibian itinerant exhibition was displayed for one month in each participating school, contributing to inform and engage the school community in the conservation of ponds and this less appreciated group.

The students participated in a pre- and post-project survey and the data collected was used to test the null hypothesis of no significant difference between the two surveys concerning the participant’s overall environmental consciousness, their attitudes towards ponds as a habitat, and their attitudes towards the ponds associated biodiversity.

In addition, the study aimed to obtain insight on using ponds as an environmental education strategy based on long-term project implementation and hands-on activities.

## Materials and Methods

### Project implementation

The project was implemented during the 2013–2014 scholar year with 264 students from eight schools from different cities of Central and North Portugal, of which six schools corresponding to 134 students participated in all project activities and evaluation. The project team performed five visits to each school and developed eight activities, including lectures, support sessions to adopt or construct a pond, to manage and monitor ponds, to develop a field activity and a classroom practical activity, to organize and install an amphibian itinerary exhibition and to train students as animal keepers for this event.

The lectures included three themes related with ponds and associated biodiversity: the first introduced the pond habitat, its importance, conservation status and biodiversity; the second addressed amphibian and reptile conservation and was held during the itinerary amphibian exhibition on display in the school; and the third lecture was about ongoing scientific research in ponds, in genetics, evolution and biodiversity conservation.

The amphibians’ itinerary exhibition “Anfíbios—uma pata na água, outra na terra” (“Amphibians—a paw on the water, another on land”) aimed to develop awareness about this group of animals. This exhibition included roll-up informative panels and terrariums with live autochthone amphibians representing the two main taxonomic orders, frogs (Anura) and salamanders (Caudata). The students participating in the project were responsible for maintaining the exhibition, feeding and monitoring the animals under the supervision of their teachers/tutors.

### Project evaluation

The evaluation consisted of pre- and post-project surveys, including the same set of questions. Both surveys were anonymous and included sociodemographic questions about the age and sex of the participants, a group of true/false questions concerning their knowledge about ponds and associated biodiversity and Likert scale groups of questions. One was about attitudes towards specific biodiversity groups shown through photos (frogs, salamanders, turtles, other reptiles, odonata, other macroinvertebrates and plants), the same in the two surveys. Another was about attitudes towards ponds, also appreciated through photographs. In these two groups, the Likert scale used five categories, from “totally dislike” (coding value 1) to “totally like” (coding value 5) with a central response of “indifferent” (coding value 3). Another two groups of Likert scale questions also addressed attitudes towards ponds and amphibians, but adapting the basic attitudes about the environment and biodiversity described by Kellert, broken into the following nine categories: aesthetic, dominionistic, ecologistic, humanistic, moralistic, naturalistic, negativistic, scientistic and utilitarian [23–25] (Table 1). Each statement was attributed a five category option response, as in the previous cases, from “totally disagree” to “totally agree”. The fifth group of Likert scale questions concerned environmental consciousness, as defined by the revised New Environmental Paradigm scale (NEP) described by Dunlap [26–28], with answers also coded in five categories, from most negative (code value 1) to most positive opinion (code value 5). The NEP scale was used in order to avoid controversial opinions on how to address this issue. The pre-project survey also included multiple-choice questions about previous knowledge and contact with ponds. The survey questions are available as supplementary material (S1 Appendix).

**Table 1:** Kellert basic attitudes adapted to ponds and amphibians.

Kellert basic attitudes	Survey sentences	
	Amphibians	Ponds
Aesthetic	I think that amphibians are very attractive living beings.	A pond makes a landscape ugly.
Naturalistic	I usually spend my free time exploring places where amphibians live, as ponds or streams.	Knowing a pond is important to learn things about nature that are not available in books.
Dominionistic	We must live in harmony with amphibians because they are important to nature’s balance.	Ponds should be drained in order to stabilize the land to urban construction or agriculture.
Ecologistic	I want to understand the relationships between amphibians, their environment and the species with which they relate.	The pond is an essential habitat to several species.
Humanistic	I really like amphibians	I like a lake with a fountain and water lilies better than natural pond.
Scientific	I am interested in knowing the physical characteristics of amphibians, the types of amphibians that exist and how their body works.	Ponds have an essential role in the planet's water cycle.
Utilitarian	I find it important to use amphibians in agriculture to feed on harmful insects.	The ponds are important to collect water for agriculture uses.
Moralistic	I am interested in amphibians and to help them not being abused by people.	The pond is a natural habitat and therefore should not be disturbed by anything or anyone.
Negativistic	I have no interest in amphibians because they never raised my curiosity.	Ponds are unpleasant because they have mosquitoes that carry human diseases.

Data from the surveys were provided and analyzed anonymously and, apart from the age and sex of the participant, surveys only included questions focusing the study objectives. The school boards and professors approved the evaluation strategy prior to the project implementation. Oral consent to use the data for scientific purposes was given by the participants and their teachers after a member of the project team read the survey header indicating the study objective.

### Data analysis

Despite the five Likert scale optional responses being categories, they were coded as values from 1 to 5 and treated as quantitative in order to calculate descriptive statistics showing the change in the students’ response from pre- to post-project. Nevertheless, the formal hypothesis test to compare pre- versus post-project responses respected the categorical nature of the Likert scale. The five optional responses to each question were coded as presence-absence variables (values 1 or 0) and the presence (value 1) attributed to the variable representing the category selected by the student. As an example, if question 3 was replied by a student with the option 4, the question was organized as variables 3.1 to 3.5 and the presence attributed to 3.4. The responses were organized in a data matrix, with the students as samples and their answers as variables. A resemblance matrix among samples was obtained using the Jaccard similarity coefficient. This matrix was further simplified by calculating the centroid or centre of gravity, of each group of students per school and time period (pre- and post-project). The centroids matrix was submitted to ordination analysis using non-metric multidimensional scaling (NMDS), and tested for the null hypothesis of no significant difference between the pre- and post-project, using a one-way Analysis of Similarities (ANOSIM). ANOSIM produces the statistic R, which relates the within group to the between groups similarities. It varies from -1 to +1 and is equal to +1 when all the replicates of the same group are more similar to each other than any of the replicates from different groups, rejecting the null hypothesis. R approaches the value 0 when the null hypothesis is true. The R statistic is accompanied by a significance value obtained by calculating the probability of the observed R within a series of R values obtained by permutation. In this case, with two groups being compared (pre- versus post-project), each with six replicates (the centroids representing the students from the six schools that completed the assessment), there were a maximum of 462 permutations, allowing to reject the null hypothesis at p = 0.002, if the observed R was larger than any of the simulated R-values from permutations (1 out of 463 = 0.002). All data analysis was conducted with the software PRIMER v6 with the add-on PERMANOVA+ [29].

The null hypothesis of no significant difference between pre- and post-project surveys was tested separately for the groups of questions dedicated to evaluate the general attitude about ponds as a habitat (H_0_1), the attitudes towards the ponds biodiversity (H_0_2), the Kellert adopted attitudes about ponds (H_0_3), the Kellert adopted attitudes toward amphibians (H_0_4), and the environmental consciousness of the participants (H_0_5). Upon rejection of the null hypothesis H_0_2, including all taxonomic groups, the test was run separately with each biodiversity group to verify for which the pre- and post-project responses differed significantly.

## Results

From a total of 264 students that participated in the project, 202 pre-project and 131 post-project valid responses were obtained, given that not all the students completed the whole set of activities. All relevant data are within the paper and its Supporting Information files. The data matrixes from the pre-project and post-project valid responses are available as supplementary material (S2 Appendix).

The students who answered surveys were between 15–18 years old. The average age on the pre-project questionnaires was 16 years old on the pre-project and 17 years old on the post-project (most students celebrated their birthday during the period of the project implementation). Considering the gender, in the pre-project questionnaires 63% of the participants were girls and 37% were boys while in the post-project the percentages were 66% and 34%, respectively.

About 80% of the students were acquainted with the pond habitat before attending the project activities. However, previous contacts with ponds were mainly indirect, such as through the Internet (62%), books or journals (61%), television (44%) or other media. Pre-project direct contact was through visits to ponds during school activities (52%) or walks in nature (50%).

The questions dedicated to assess prior knowledge about ponds and associated biodiversity showed that students answered correctly 60% of the pre-project questions, of which 67% related to pond ecology subjects and 52% to identify biodiversity correctly. In the post-project questionnaires 66% responses were correctly answered, which corresponded to a significant improvement (*χ*^2^ = 17.696; *p* ≤ 0.0001). The percentage of correct answers related to pond ecology was still higher (73%) than those related to pond biodiversity (60%), but the increase was larger in the latter (8%).

Table 2 summarizes pre- versus post-project Likert scale mean values as well as the ANOSIM R-statistic values and associated significance considering the various null hypothesis and questions.

**Table 2:** Likert scale mean values and ANOSIM R-statistic with the associated significance for the comparison between pre- and post-project surveys. ns = non significant.

Variables	Pre-project Likert scale mean value	Post-project Likert scale mean value	ANOSIM	
			R-statistic	Significance (p)
Attitudes towards Ponds (H_0_1)	3.48	4.02	0.465	0.006
Attitudes towards Biodiversity (H_0_2)	3.13	3.60	0.409	0.002
Frogs and toads	3.06	3.60	0.435	0.004
Salamanders and newts	2.75	3.67	0.657	0.002
Turtles	3.98	4.11	-0.039	0.589 ns
Snakes and lizards	2.59	3.15	0.244	0.058 ns
Dragonflies	2.97	3.43	0.116	0.160 ns
Other macroinvertebrates	2.58	3.10	0.131	0.130 ns
Plants	4.03	4.17	-0.165	0.996 ns
Kellert basic attitudes towards Ponds (H_0_3)	3.09	3.24	0.233	0.041
Kellert basic attitudes towards Amphibians (H_0_4)	3.22	3.57	0.263	0.022
Environmental consciousness (H_0_5)	3.60	3.56	-0.017	0.550 ns

**Fig 1:**
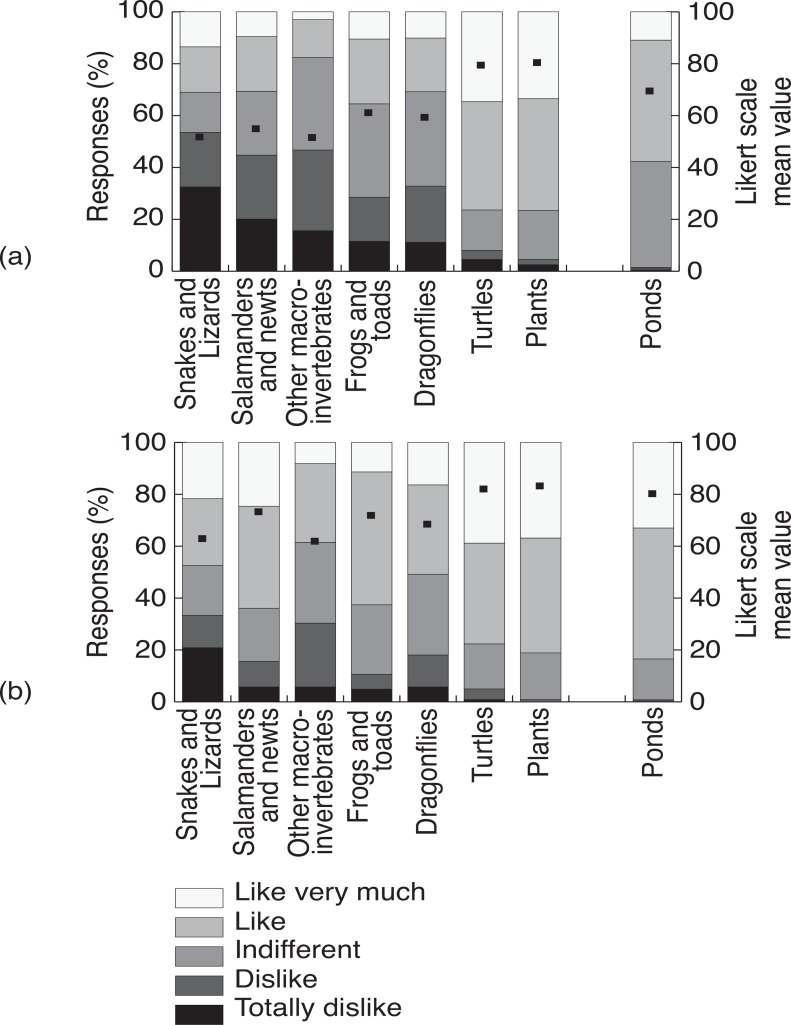
Likert scale response categories in (A) pre- and (B) post-project surveys responses regarding attitudes towards the various biodiversity groups and the pond habitat. Likert scale mean values are indicated over each bar as a black square symbol (left axis).

**Fig 2:**
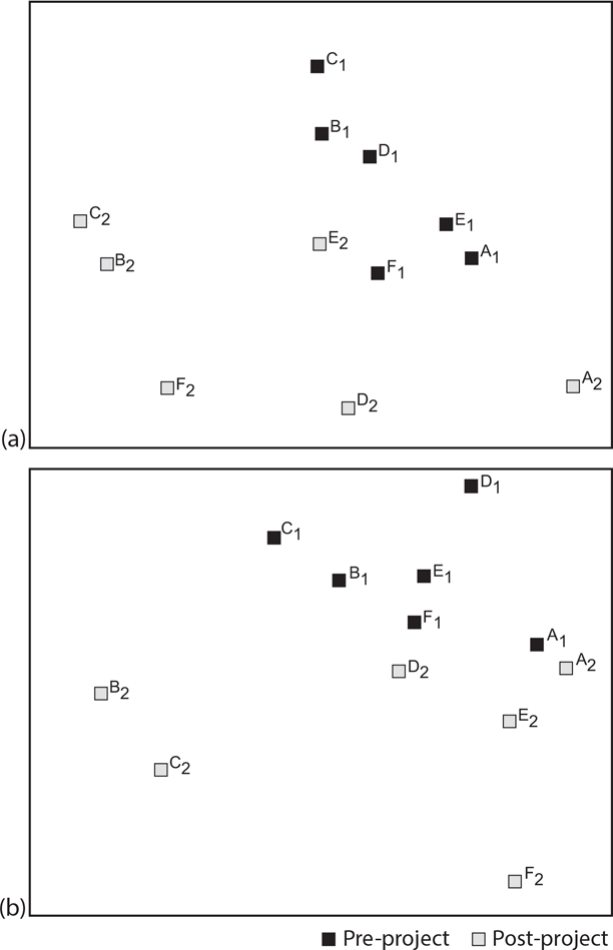
Ordination diagrams (NMDS) representing the schools centroids (A-F) for the pre- and post-project responses (1 and 2, respectively), relative to the attitudes towards ponds as a habitat (a) and their associated biodiversity (b).

The attitudes towards ponds as habitats and their associated biodiversity, as well as the Kellert basic attitudes towards ponds and specifically towards amphibians, all changed significantly from pre- to post-project, rejecting null hypotheses H_0_1to H_0_4 as indicated in Table 2 and illustrated in Figs 1 and 2.

Concerning the attitudes towards the biodiversity groups, null hypothesis H_0_2, the overall significant improvement noticed from pre- to post-project surveys was not generalized to all the groups, as shown by the statistical significance associated with the individual biodiversity groups test results presented in Table 2. Only the amphibian groups rejected the null hypothesis, the largest difference from pre- to post-project survey being for the Salamanders and Newts, as indicated in Table 2 through the highest value of the R-statistic or the biggest change in the Likert-scale coded mean value. No other individual biodiversity group rejected the null hypothesis, either because the environmental education action was not directed to them, namely the case of the snakes or lizards (cf. Table 2 and Fig 1), or because the group was already highly appreciated by the students before the project, as was the case of the turtles or the plants (cf. Table 2 and Fig 1).

No change was detected in the environmental consciousness of the students as a result of their participation in the project. The null hypothesis H_0_5 was not rejected in the ANOSIM test and the Likert-scale coded mean values were almost the same in the pre- and post-project surveys, as shown in Table 2. A brief analysis of the responses related to environmental consciousness, the NEP scale showed that the students believe in the human capacity to solve environmental problems and intelligently exploit new natural resources and also in the impact of human activities in nature and the environment.

## Discussion

This study showed that implementing the environmental education action “Choose Science—Ponds with Life” during one school year modified the knowledge and attitudes towards ponds and their biodiversity, particularly amphibians, in high school students aged 15 to 18.

The students’ pre-project contact with ponds was mainly indirect, through Internet, television or books. Other authors already recognized that contact with nature is becoming more dependent of indirect pathways as media [22, 30]. Direct contact was moderately common and limited to walks in nature or to existing ponds in schools. Given the significant outcomes of the project, previous direct contact with ponds must have been mainly limited to nature appreciation.

The number of correct answers about ponds and their biodiversity was higher in the post- than in the pre-project survey, indicating a limited knowledge about those species. Although knowledge cannot be considered a vehicle to changing attitude, authors have sustained that it facilitates such changes [31, 32]. Knowledge acquired in environmental education programs may also not last long and projects solely based on knowledge acquisition may not be as efficient in driving attitude changes. The project “Choose Science—Ponds with Life” was a long-term project, extending over one full scholar year and engaged the students in a range of different activities which promoted their personal responsibility on the success of the outcomes, such as the set-up and follow-up of a pond or the preparation and delivery of the live amphibian exhibition. Such a mixture of activities has the potential to maintain the acquired knowledge for longer and thus effectively contribute to attitude development/change.

The present study used an innovative approach to compare pre- and post-project surveys, assuming the non-quantitative nature of the Likert-scale optional categorical responses and coding them as presence-absence variables. This allowed building a similarity matrix among students solely on the patterns of their responses that was then analyzed by multivariate methods developed for ecological research but that can easily be applied to other studies. The results reported here show the method was very efficient to compare pre- and post-project surveys and that it can be easily suited for the evaluation of environmental education/science communication projects, providing social researchers with an effective multivariate hypothesis testing tool, uncommonly used in the social sciences.

The student attitudes towards ponds as habitats improved significantly over the course of the project. This was shown by the Likert-scale coded mean values, the Kellert adopted attitude values and the statistical significance associated with the hypothesis tests. The same was observed for the biodiversity associated with the ponds, but in this case the changes from pre- to post-project were not consistent across all biodiversity groups. The significant changes were noted only in the two amphibian groups, frogs and salamanders. Other authors also noticed that amphibians can commonly be neglected or even negatively connoted by the public but have great potential to engage the public in science education activities and foster positive changes, given their biological characteristics, among which their morphological variety and adaptation to aquatic habitats, reproduction, larval and metamorphosis phases easily observed in water bodies close to urban areas [19, 33, 34]. The temporary exhibition about amphibians and the fact that most of the lectures, classroom and field activities were associated with this group also facilitated the observed changes towards this group of animals.

Differences in student attitudes towards Odonata and other macroinvertebrates were not statistically significant from pre- to post-project. Despite invertebrates can be easily found and observed in ponds, their characteristics may be important barriers for environmental education and attitude changes, as mentioned by other authors [25, 35]. A morphology very different from human [25, 36], cultural heritage associating invertebrates with danger and the spread of diseases [37, 38] and phylogenetic distance from humans that culminates in a very different morphology and behaviour [39, 40], all have been considered to foster an overall negative human attitude towards invertebrates.

Scaled reptiles also did not show significant differences between pre- and post-project surveys, although the statistical significance associated with the R-statistic was borderline (p = 0.06, cf. Table 2). The fact that these animals were rarely seen could have contributed to this result but also cultural heritage might be responsible for negative attitudes towards reptiles. This is in agreement with authors who sustain that without intensive educational actions people may not be prepared to protect this group of animals [41–44]. Turtles also did not show significant differences between the two assessment periods, but contrary to snakes and lizards, were already appreciated by students before the project implementation. Other authors also described better attitudes towards turtles when compared to other reptiles, namely because turtles are adopted as pets, have no venomous species and often show positive connotations in books and media [19, 35, 45, 46].

Plants were also among the most appreciated biodiversity groups in the pre-project phase as demonstrated by the high Likert-scale coded mean value of the group and again did not reject the null hypothesis. Other authors have shown overall positive attitudes from the public towards plants even if plant are generally seen lifeless or even worthless [47, 48].

The environmental consciousness of the participants did not change significantly due to the implementation of the project and kept a medium-good level in the Likert-scale coded mean value, according to the NEP scale [27]. The pre-project questionnaires showed that students were already environmentally conscious particularly regarding human impacts in nature and the environment. A closer analysis showed that many shared the belief that humankind is able to solve any environmental problem and intelligently exploit new natural resources. Similar results have been reported by other authors, as well as the fact that, in most of the public mental concepts of nature, humankind is seen as set apart from nature and having a separate species condition [39, 49]. This also indicates that a medium-good environmental consciousness may not translate into attitudes or behaviour towards a better environment [31, 32, 50–54].

Overall, these results indicated that the environmental education strategy proposed in this work had important outcomes in education and attitudes towards biodiversity and the environment, especially when considering amphibians and other groups of species that can be easily observed and manipulated [33, 50, 55–58].

## Conclusion

As a general conclusion, this study showed that environmental education actions based on direct contact are able to modify public attitudes towards biodiversity, namely amphibians. In addition, ponds proved to be a good habitat model from an educational point of view as they allowed a variety of outdoor hands-on exploration activities about habitat and ecological functioning. Ponds efficiently promoted a direct contact with nature and life forms, including flagship and bio-indicator species, in urban areas and schools gardens. Although small in size, ponds promoted an holistic view about ecosystem structure and function, ecological succession, relationships between species and management through conservation strategies.

## Supporting Information

S1 Appendix Likert scale questions equally used in the pre- and post-project surveys for the analysis of the five null hypotheses mentioned in the text. (DOCX)

S2 Appendix Data matrixes from the pre-project and post-project valid responses. (XLSX)
