# Supplementary material for: Can Environmental Education Actions Change Public Attitudes? An Example Using the Pond Habitat and Associated Biodiversity
Source: PLoS One. 2016 May 5;11(5):e0154440. doi: 10.1371/journal.pone.0154440 (PMC4858173; doi:10.1371/journal.pone.0154440)
Supplement: S1 Appendix — (DOCX) [file pone.0154440.s001.docx]

**S1 Appendix:**

**S1 Appendix:** Likert scale questions equally used in the pre- and post-project surveys for the analysis of the five null hypotheses mentioned in the text.

|  |  |  | | | | |
| --- | --- | --- | --- | --- | --- | --- |
|  |  | I dislike very much | I dislike | I am indifferent | I like | I like very much |
| Attitude towards ponds | What is your opinion about ponds? | **□** | **□** | **□** | **□** | **□** |
| Attitude towards biodiversity groups | Look at the biodiversity groups presented in the projection. Mark your opinion about the organism of each biodiversity group. | **□** | **□** | **□** | **□** | **□** |
| Attitude towards ponds (Kellert basic attitudes) | Mark your opinion about the following sentences | Totally disagree | Disagree | I am indifferent | Agree | Totally agree |
|  | A pond makes a landscape ugly. | **□** | **□** | **□** | **□** | **□** |
|  | Knowing a pond is important to learn things about nature that are not available in books. | **□** | **□** | **□** | **□** | **□** |
|  | Ponds should be drained in order to stabilize the land to urban construction or agriculture. | **□** | **□** | **□** | **□** | **□** |
|  | The pond is an essential habitat for several species. | **□** | **□** | **□** | **□** | **□** |
|  | I like a lake with a fountain and water lilies better than natural pond. | **□** | **□** | **□** | **□** | **□** |
|  | Ponds have an essential role in the planet's water cycle. | **□** | **□** | **□** | **□** | **□** |
|  | The ponds are important to collect water for agriculture uses. | **□** | **□** | **□** | **□** | **□** |
|  | The pond is a natural habitat and therefore should not be disturbed by anything or anyone. | **□** | **□** | **□** | **□** | **□** |
|  | Ponds are unpleasant because they have mosquitoes that carry human diseases. | **□** | **□** | **□** | **□** | **□** |
| Attitude towards amphibians (Kellert basic attitudes) | Mark your opinion about the following phrases | Totally disagree | Disagree | I am indifferent | Agree | Totally agree |
|  | I think that amphibians are very attractive living beings. | **□** | **□** | **□** | **□** | **□** |
|  | I usually spend my free time exploring places where amphibians live, as ponds or streams. | **□** | **□** | **□** | **□** | **□** |
|  | We must live in harmony with amphibians because they are important to nature’s balance. | **□** | **□** | **□** | **□** | **□** |
|  | I want to understand the relationships between amphibians, their environment and the species with which they relate. | **□** | **□** | **□** | **□** | **□** |
|  | I really like amphibians | **□** | **□** | **□** | **□** | **□** |
|  | I am interested in knowing the physical characteristics of amphibians, the types of amphibians are and how their body works. | **□** | **□** | **□** | **□** | **□** |
|  | I find it important to use amphibians in agriculture to feed on harmful insects. | **□** | **□** | **□** | **□** | **□** |
|  | I am interested in amphibians to and help them not being abused by people. | **□** | **□** | **□** | **□** | **□** |
|  | I have no interest in amphibians because they never raised my curiosity. | **□** | **□** | **□** | **□** | **□** |
| Environmental consciousness (Dunlap, 2010 NEP scale) | Mark your opinion about the following phrases | Totally disagree | Disagree | I am indifferent | Agree | Totally agree |
|  | We are approaching the limit of the number of people the earth can support. | **□** | **□** | **□** | **□** | **□** |
|  | Humans have the right to modify the natural environment to suit their needs. | **□** | **□** | **□** | **□** | **□** |
|  | When humans interfere with nature it often produces disastrous consequences | **□** | **□** | **□** | **□** | **□** |
|  | Human ingenuity will insure that we do not make the earth unlivable | **□** | **□** | **□** | **□** | **□** |
|  | Humans are severely abusing the environment | **□** | **□** | **□** | **□** | **□** |
|  | The earth has plenty of natural resources if we just learn how to develop them | **□** | **□** | **□** | **□** | **□** |
|  | Plants and animals have as much right as humans to exist | **□** | **□** | **□** | **□** | **□** |
|  | The balance of nature is strong enough to cope with the impacts of modern industrial nations | **□** | **□** | **□** | **□** | **□** |
|  | Despite our special abilities humans are still subject to the laws of nature | **□** | **□** | **□** | **□** | **□** |
|  | The so-called “ecological crisis” facing humankind has been greatly exaggerated | **□** | **□** | **□** | **□** | **□** |
|  | The earth is like a spaceship with very limited room and resources | **□** | **□** | **□** | **□** | **□** |
|  | Humans were meant to rule over the rest of nature | **□** | **□** | **□** | **□** | **□** |
|  | The balance of nature is very delicate and easily upset | **□** | **□** | **□** | **□** | **□** |
|  | Humans will eventually learn enough about how nature works to be able to control it | **□** | **□** | **□** | **□** | **□** |
|  | If things continue on their present course, we will soon experience a major ecological catastrophe | **□** | **□** | **□** | **□** | **□** |

Note: the questionnaire was accompanied by the projection of images of ponds and of the different biodiversity groups.
